# Systemic TGF-β1 reduction contributes to neuronal GLUT4 trafficking impairment in acute hepatic encephalopathy

**DOI:** 10.3389/fnmol.2026.1849470

**Published:** 2026-06-10

**Authors:** M. Popek, M. Zielińska

**Affiliations:** Department of Neurotoxicology, Mossakowski Medical Research Institute, Polish Academy of Sciences, Warsaw, Poland

**Keywords:** AMPK, glucose, GLUT4, hepatic encephalopathy, TGF-β1

## Abstract

Acute liver failure disrupts cerebral glucose homeostasis and contributes to neurological manifestations of hepatic encephalopathy (HE). Neurons, which partly rely on the insulin-sensitive glucose transporter GLUT4, may be particularly vulnerable to systemic metabolic disturbances. Transforming growth factor beta 1 (TGF-β1), implicated in HE pathogenesis, is known to modulate insulin signaling in peripheral tissues; however, its role in neuronal glucose handling remains poorly defined. In this study, we investigated whether reduction of peripheral TGF-β1 in HE impairs the neuronal GLUT4 status and whether this effect can be reproduced by systemic TGF-β1 neutralization (sTGF-β1n) in healthy mice. Both HE and sTGF-β1n increased GLUT4 immunoreactivity in MAP2-positive neurons and promoted its accumulation within the neuronal cytoplasm. Together with unaltered GLUT4 membrane levels, this suggests defective cytosol-to-membrane translocation or mobilization of the transporter. These changes were accompanied by altered PI3K/Akt/PKCζ signaling in HE only, but decreased AMPK phosphorylation in both model treatments, indicating the latter route to be the common mediator of impaired regulation of GLUT4 trafficking. While hypoglycemia and hyperinsulinemia were observed only in the HE model, selective sTGF-β1n reproduces alterations in neuronal GLUT4 distribution in the absence of hypoglycemia and hyperinsulinemia accompanying HE in the present model, indicating that TGF-β1 deficiency *per se* may contribute to this deficit. Our findings highlight TGF-β1 availability as a previously underappreciated modulator of neuronal glucose metabolism, and peripheral reduction of TGF-β1 as a factor potentially aggravating energy deficit in HE.

## Graphical Abstract

**Figure:**
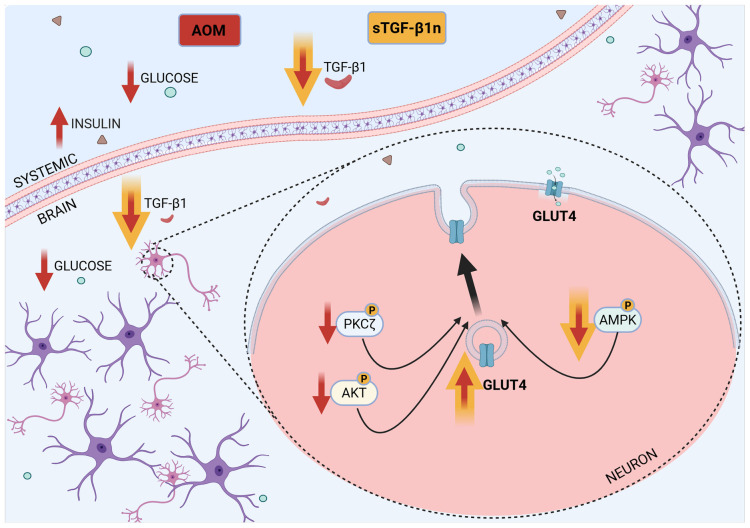
TGF-β1–dependent and independent metabolic alterations in hepatic encephalopathy: common markers identified in two experimental models. The graphical abstract illustrates peripheral and brain alterations in glucose metabolism parameters observed in two experimental models: acute hepatic encephalopathy (AOM) model and systemic TGF-β1 neutralization (sTGF-β1n) model. Arrows indicate the direction of change (upregulation/downregulation) in specific parameters. Red arrows represent changes unique to the AOM model, while orange arrows indicate changes specific to the sTGF-β1n model. Overlapping red-orange arrows highlight shared alterations, suggesting common molecular mechanisms. These common changes may reflect a potential role of TGF-β signaling in the metabolic dysfunction associated with hepatic encephalopathy. Created in BioRender. Szumska, K. (2025) https://BioRender.com.

## Highlights

- Peripheral and brain TGF-β1 levels are reduced in acute hepatic encephalopathy (HE).
- Low glucose level is observed in acute HE model.
- HE and TGF-β1 neutralization raise cytosolic GLUT4 and reduce AMPK phosphorylation.
- TGF-β1 contributes to the regulation of neuronal energy metabolism in acute HE.

## 1 Introduction

Acute liver failure alters cerebral glucose handling, as the effect of fluctuations in blood glucose levels and elevated insulin resulting from impaired hepatic clearance, reported both clinically and in HE animal models (Garcia-Compean et al., 2009; Hung et al., 2021; Nogueira and Cusi, 2024; Prasad et al., 2024). This consequence of liver impairment significantly contributes to the development of neurological symptoms (Hung et al., 2021; Li et al., 2021). Accumulation of various toxins in the bloodstream, with neurotoxic ammonia being the most extensively studied (Duarte et al., 2024; Prasad et al., 2024), contributes to central metabolic and energy disturbances (Rama Rao and Norenberg, 2012; Prasad et al., 2024). Such alterations may lead to insulin resistance, a condition that can further disrupt cerebral glucose metabolism. Since neurons rely on a tightly regulated supply of glucose, insulin insensitivity may affect the function and localization of key glucose transporters. Available evidence points to selective changes in transporters localized on the blood-brain-barrier forming endothelial cells and astrocytes rather than clear data about insulin-dependent neuron-specific transporters; in experimental ALF, endothelial and astrocytic GLUT1 expression and cortical glucose uptake increase, whereas neuronal GLUT3 remains unchanged, implicating non-neuronal cells’ adaptation to systemic metabolic stress (Bélanger et al., 2006). However, data directly documenting changes in insulin-dependent transporters (e.g., GLUT4) in neurons upon ALF are insufficiently evidenced.

Thus, in this study, our attention has been drawn to GLUT4, an insulin-responsive glucose transporter known to be expressed in neurons (Ashrafi et al., 2017), whose proper translocation to the plasma membrane is critical for maintaining glucose homeostasis under metabolic stress.

It is well-known that systemic factors may modulate neuronal metabolism (van Dijk et al., 2015; Rae et al., 2024). Among these, transforming growth factor beta 1 (TGF-β1), a pleiotropic cytokine implicated in HE pathomechanism, has been proposed to influence brain glucose utilization. TGF-β1 is a multifunctional cytokine involved in numerous regulatory pathways (Deng et al., 2024), including those related to glucose metabolism and insulin signaling (Wu and Derynck, 2009; Liu and Chen, 2022; Pan et al., 2023). Altered TGF-β1 levels have been observed in various HE models, and evidence suggests that peripheral changes in this cytokine concentration may influence its cerebral levels (McMillin et al., 2014, 2019; Fabregat et al., 2016; Popek et al., 2022). Liver dysfunction has been shown to affect hepatic TGF-β1 expression, and conditions such as thrombocytopenia, frequently observed in HE patients and in experimental models (Schiødt et al., 2003; Hancox and Smith, 2013; Popek et al., 2022), have been associated with reduced circulating levels of this cytokine. The role of TGF-β1 in modulating insulin-sensitive glucose uptake has been documented in peripheral tissues, including adipocytes and muscle cells (Hahn et al., 2020; Toyoda et al., 2022; Phuong et al., 2025), and its regulatory effects have also been highlighted in animal models of diabetes (D’Alessandro et al., 2022). Notably, TGF-β1 appears to influence key components of the insulin signaling pathway, such as the insulin receptor and downstream effectors, suggesting its potential role in the central regulation of glucose homeostasis.

**FIGURE 1:**
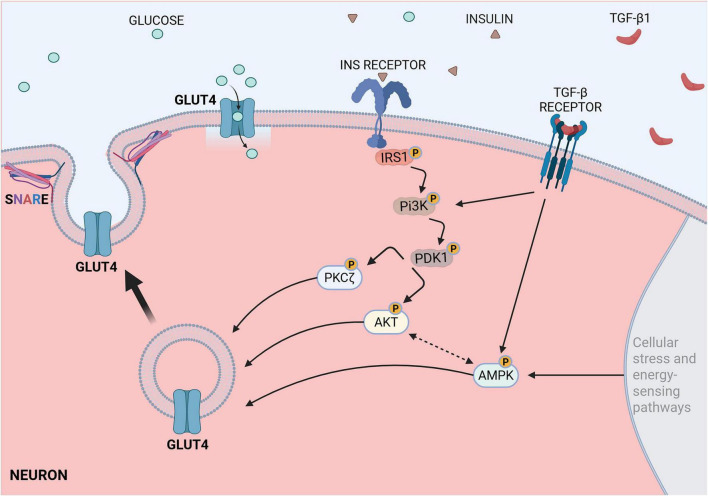
Canonical insulin signaling, AMPK-associated signaling, and the non-canonical TGF-β1 pathway converge to promote neuronal GLUT4 translocation via SNARE-mediated membrane fusion. Schema was created using BioRender.com.

The present study aims to investigate whether a peripheral reduction in TGF-β1 levels observed in a mouse model of acute HE and reflected in the corresponding cerebral level could contribute to alterations in insulin-dependent glucose transport in neurons. This research seeks to expand our understanding of the liver-brain axis in acute HE and identify potential modulators of glucose metabolism beyond the toxic effect of ammonia. Specifically, we will focus on the molecular aspects of GLUT4 transporter translocation to the neuronal membrane, particularly the PI3K/Akt/PKCζ signaling pathway, as well as an alternative pathway that engages an AMPK-mediated, insulin-independent mechanism (Figure 1).

## 2 Materials and methods

### 2.1 Animal models

All animal experiments were conducted in accordance with the approval of the local ethics committee in Warsaw (498/2017) following EC Directive 86/609/EEC. The acute liver injury model was established by a single intraperitoneal injection of the hepatotoxin azoxymethane (AOM) at a dose of 100 mg/kg. In a separate experimental group, systemic TGF-β1 neutralization (sTGB-β1n) was induced by a single intraperitoneal administration of a TGF-β1/1.2 neutralizing antibody (1 mg/kg b.w., cat. no. AB-101-NA; R&D Systems, a Bio-Techne brand, United States). Control mice for both models received an equivalent volume of physiological saline. All experiments were performed on male C57BL/6J mice aged 3–4 months, obtained from the animal facility of the Mossakowski Medical Research Institute, Polish Academy of Sciences (MMRI PAS), to ensure group consistency and to avoid potential sex-related variability, which was not the focus of this study.

All animals were housed in individually ventilated cages under standard conditions (12-h light/dark cycle) with unrestricted access to food and water. Mice in the AOM model were monitored more closely; beginning 8 h post-injection, they were provided with a heating pad to prevent hypothermia. They were also hydrated with glucose-supplemented saline at 8 and 16 h post-injection.

At the experimental endpoint (22 h post TGF-β1/1.2 neutralizing antibody administration and around 24 h after AOM injection), animals were euthanized by decapitation under deep isoflurane anesthesia (4% isoflurane in an oxygen/air mixture, administered for approximately 1 min until loss of reflexes). Tissues were immediately collected either into dry ice or an appropriate buffer, depending on the downstream experimental protocol.

### 2.2 Behavioral assessments

Mice were habituated to the experimental arena (30 × 37 cm) for 5 min on the day preceding the experiment in the absence of objects. In the experimental session, 18–19 h after intraperitoneal injection, mice were subjected to the Novel Object Recognition (NOR) test. During the familiarization phase, animals were allowed to explore two identical objects (A × A) for 5 min. After a 1 h interval, mice were re-exposed to the arena for 5 min in the presence of one familiar and one novel object (A × B).

Object exploration was defined as nose-directed investigation of the object at close proximity. Exploration time (excluding time spent sitting on objects) was recorded as total object exploration (A + B) and novel object preference (% of total); familiarization phase parameters (A vs. A’) are provided in Supplementary Material 1. Based on previous literature, animals with total exploration time below 10 s during the familiarization phase were excluded from NOR analysis due to insufficient exploratory activity. Although the AOM-treated group did not meet the minimal exploratory activity threshold required for quantitative NOR analysis, the corresponding raw behavioral data are shown in the figure for transparency and to provide context for the observed inability to perform the task.

Two hours after completion of the NOR procedure, mice were subjected to a 5 min Open Field test in a separate arena (50 × 50 cm) to assess locomotor activity and anxiety-related behavior as control parameters for NOR interpretation.

### 2.3 Documentation of peripheral tissue morphology

The liver, kidney, and pancreas were isolated immediately after decapitation of the animals. Tissue samples approximately 0.5 × 0.5 cm in size were placed into Eppendorf tubes and gradually cooled down to −80 °C for preservation.

Subsequently, the tissues were sectioned using a cryostat (model: Leica CM1860 cryostat) at −23 °C. Sections with a thickness of 30 μm were mounted directly onto glass microscope slides. Histological staining with hematoxylin and eosin (H&E) was then performed according to standard protocols. Briefly, the tissue sections were rehydrated through a graded alcohol series and rinsed in distilled water. Next, they were stained with hematoxylin for 5 min, followed by rinsing under a gentle stream of tap water for 12 min. Counterstaining with eosin was performed for 3 min. After a quick rinse in low-percentage alcohol, the sections were dehydrated in a graded alcohol series and mounted using DPX mounting medium (Sigma-Aldrich, 06522, Poznań, Poland) under a coverslip.

Images were captured using a Olympus IX71 Inverted Fluorescence Motorized Microscope (Olympus Corporation, Tokyo, Japan) at magnifications of × 100 and × 400. All sections from each of the three mice in every experimental group were analyzed to ensure they exhibited similar morphological characteristics, which are described in detail later in this study.

### 2.4 Measurement of biochemical parameters in blood

Blood was collected immediately after decapitation into lithium-heparin-coated microtubes (LH 1.3 Micro tube; SARSTEDT, Germany). Within 5 min, the blood samples were centrifuged at 4 °C at 3500 × *g* for 5 min. The collected plasma was then analyzed using the IDEXX Catalyst One system, which measures various biochemical parameters with ready-to-use test cartridges for glucose, urea, ALT, ALP, amylase, and lipase (IDEXX Laboratories, Inc., Westbrook, Maine, United states). For the measurement of insulin levels in blood plasma, an ELISA kit was used (Merck Sp. z o.o., EZRMI-13K, Poland). Protein concentration was determined using the BCA protein assay kit (Life Technologies Polska Sp. z o.o., 23227, Poland). To assess both the active and inactive forms of TGF-β1 in plasma, Western blot analysis was performed on plasma samples using antibodies: TGF-β1/1.2 (cat. no. AF-101-NA; R&D Systems, a Bio-Techne brand, United states), TGF-β1 (cat. no. 26155-1-AP; Proteintech Group, Inc., Rosemont, IL, United states), following the standard protocol described in the Western blot section. Quantitative results of total and active TGF-β1 levels in mouse plasma for the studied models, obtained through ELISA measurements, are available in Popek et al. (2022).

### 2.5 Preparation of homogenates and cytosolic and membrane fractions

The cerebral cortex was promptly dissected from the mice brains on ice and manually homogenized using a teflon-glass homogenizer in a buffer containing 20 mM Tris-HCl (pH 6.8), 137 mM NaCl, 2 mM EDTA, 1% Triton X-100, 0.5 mM DTT, 0.5 mM PMSF, along with phosphatase and protease inhibitor cocktails (dilutions 1:100 and 1:200, respectively). The homogenate was then centrifuged at 12,000 × *g* for 10 min.

To isolate the P2 and S2 fractions, the cortex was homogenized in a separate buffer composed of 15 mM Tris-HCl (pH 7.6), 0.25 M sucrose, 1 mM DTT, 0.5 mM PMSF, and the same inhibitor cocktails. This homogenate was centrifuged at 1,000 × *g* for 10 min at 4 °C to obtain the P1 fraction. The resulting supernatant underwent further centrifugation at 14,000 × *g* for 20 min at 4 °C, yielding the S2 fraction (supernatant), which was collected. The pellet was resuspended in a buffer and stored frozen as the P2 fraction. Protein concentrations for all samples were determined using the BCA Protein Assay Kit (Thermo Scientific, Pierce, Rockford, IL, United States).

### 2.6 Measurement of glucose and insulin levels in the brain

Previously prepared homogenates from weighed frontal cortex tissue samples obtained from mice transcardially perfused with 15 ml PBS were used for glucose and insulin measurements. Brain tissue was homogenized in PBS supplemented with phosphatase and protease inhibitors to obtain 15% homogenates. Homogenates were centrifuged at 12,000 × *g* for 15 min, and the resulting supernatants were used for further analyses. Glucose concentrations were determined using a handheld electrochemical (amperometric) detection system and normalized to tissue weight (expressed per gram of tissue).

Insulin concentrations were measured in duplicate using a Mouse Insulin ELISA Kit (cat. no. KE10089; Proteintech Group Inc., Rosemont, IL, United States), with a reported sensitivity of 1.3 pg/ml and a detection range of 6.25–400 pg/ml. Brain homogenates were diluted 1:2 in sample dilution buffer prior to analysis. To verify assay performance in the brain tissue matrix, spike-and-recovery and dilution-linearity tests were performed, demonstrating acceptable recovery and linearity. As a positive technical control, insulin measurements were additionally performed in mouse serum samples diluted 1:50 and 1:100 (Supplementary Material 1). Measured values were within the dynamic range of the assay. Results were normalized to wet tissue weight and expressed per gram of tissue.

### 2.7 Measurement of d-glucose uptake in brain slices from experimental animals

The procedure for measuring D-glucose uptake was adapted from the method reported by Lipko and Debski (2018), with modifications as detailed below. The brains from control, sTGB-β1n and AOM mice were isolated and immediately immersed in oxygenated Krebs buffer of the following composition (in mM): NaCl (150); KCl (3); CaCl2 (2); MgCl2 (0.8); glucose (5); HEPES (10), aerated with 95% O2 and 5% CO2 at pH 7.4, temp. 37 °C ± 0.5 °C. The dissected brain was cut into 350 μm thick sections.

Brain micro-slices were first pre-incubated for 30 min in standard Krebs buffer containing glucose, followed by 15 min of pre-incubation in glucose-free Krebs buffer to deplete endogenous glucose. Next, slices were incubated for 15 min in a glucose-free Krebs buffer supplemented with 1 μM insulin to stimulate insulin-dependent glucose uptake—a strategy commonly used to enhance insulin responsiveness in *ex vivo* tissues prior to uptake measurement.

After the 30-min glucose deprivation phase, glucose uptake was initiated by incubating the slices for 30 min in Krebs buffer containing 2-[1,2-^3^H(N)]-deoxy-D-glucose (PerkinElmer, Waltham, MA, United States; specific radioactivity 250 μCi (9.25 MBq); final concentration 0.25 μCi/ml) and 1 mM unlabeled D-glucose to mimic physiological glucose concentrations and assess uptake under competitive conditions. In control conditions, no insulin was added.

Following incubation, brain slices were transferred onto a perfusion system and rapidly washed three times with ice-cold glucose-free Krebs buffer (2 ml per wash) to stop uptake and remove excess extracellular isotope. Slices were then weighed for normalization purposes, placed into scintillation vials, and incubated with 2 ml of scintillation fluid. Radioactivity retained in the brain tissue (reflecting accumulated glucose analog) was measured using a Wallac 1409 liquid scintillation counter (PerkinElmer, Turku, Finland).

### 2.8 Western blot analysis

Thirty micrograms of total protein (or cytosolic/membrane fraction in the case of GLUT4 protein level analysis) were separated on a 10% SDS-polyacrylamide gel (Thermo Fisher Scientific, Waltham, MA, United States) and transferred onto a nitrocellulose membrane (Bio-Rad Laboratories, 1620112, CA, United States) using the semi-dry Trans-Blot Turbo System (Bio-Rad Laboratories, CA, United States). Membranes were blocked with 5% dry milk [or 5% bovine serum albumin (BSA) (BioShop; Lab Empire S.C.) for phospho-antibodies] and incubated overnight at 4 °C with primary antibodies diluted in 1% dry milk (or 2.5% BSA for phospho-antibodies). The following primary antibodies were used: AMPKα (cat. no. 5831S), phospho-AMPKα (Thr172, clone 40H9; cat. no. 2535S), PI3 kinase p55 (clone D2B3; cat. no. 11889S), phospho-PDK1 (Ser241, clone C49H2; cat. no. 3438), phospho-Akt (Thr308, clone 244F9; cat. no. 4056S), and Akt (cat. no. 4691S) (all from Cell Signaling Technology, United States). Anti-IRS1 (cat. no. 06-248) was obtained from Merck Group (Poland). INSRβ (cat. no. sc-57342), TGFBR2 (clone E-6; cat. no. sc-17792), PKCζ (cat. no. sc-216), and GLUT3 (cat. no. sc-74399) were purchased from Santa Cruz Biotechnology (Germany). VAMP2 (cat. no. AB3347), SNAP23 (cat. no. AB3340), STX4 (cat. no. AB184545), phospho-PKCζ (Thr560; cat. no. ab62372), GLUT4 (cat. no. ab654), were obtained from Abcam (United Kingdom). Antibody dilutions were used according to the manufacturer’s datasheets (details in Supplementary Material 1). Detection was performed using appropriate HRP-conjugated secondary antibodies and chemiluminescent substrate (GE Healthcare Amersham, Piscataway, NJ, United States). Following signal detection, membranes were stripped and reprobed with HRP-conjugated anti-GAPDH antibody (1:6000; HRP-60004, Proteintech Group, Inc., Rosemont, IL, United States) as a loading control. Chemiluminescence was captured using the G-Box imaging system (SynGene). Densitometric quantification was performed with ImageJ software (NIH, Bethesda, MD, United States), on the original images, and all numerical datasets underlying the analyses are available upon reasonable request; representative full-length blots are provided in the Supplementary Material 1.

### 2.9 Immunohistochemical procedure and data analysis

The brains of control, sTGB-β1n, and AOM mice were rapidly removed, immediately frozen in powdered dry ice, and stored at −80 °C. Frontal cortex sections (20 μm thick) were prepared using a Leica CM1860 cryostat and processed according to a standard immunofluorescence protocol. Briefly, sections were fixed in 4% paraformaldehyde (PFA) for 20 min, then blocked for 1 h in 10% normal goat serum diluted in 0.1% Triton X-100. This was followed by overnight incubation with one of the following primary antibodies: phospho-AMPKα (Thr172; cat. no. 2535S; Cell Signaling Technology, United States), phospho-Akt (Thr308; cat. no. 4056; Cell Signaling Technology, United States), phospho-PKCζ (Thr560; cat. no. ab62372; Abcam, United Kingdom) or GLUT4 (1:150; cat. no. ab654; Abcam, United Kingdom). The next day, sections were incubated for 1 h with a goat anti-rabbit IgG Alexa Fluor 488 secondary antibody (1:500; Life Technologies, Carlsbad, CA, United States).

For neuronal labeling, anti-Neurofilament 200 (phosphorylated and non-phosphorylated; cat. no. N0142; Sigma-Aldrich, United States) or MAP2 was used as a neuronal marker (cat. no. MA5-12826, Life Technologies, Carlsbad, CA, United States), and a goat anti-mouse IgG Alexa Fluor 546 secondary antibody (1:500; Life Technologies, United States) were used. Cell nuclei were counterstained with Hoechst 33258 (5 μM; 1:500; Life Technologies, United States). Negative controls were processed in parallel, omitting the primary antibodies.

Z-stack images (∼10–15 μm in the z-dimension) were acquired using a confocal laser scanning microscope (LSM 780, Carl Zeiss Meditec, Germany) under identical laser power, gain, and offset settings across all experimental groups. Immunofluorescence image analysis was performed using ZEN software (v.2008, Carl Zeiss). The fluorescence intensity of phospho-AMPKα (Thr172), phospho-Akt (Thr308), and phospho-PKC zeta (Thr560) was quantified by measuring the mean gray value of maximum intensity Z-projected images in ImageJ software, and normalized to the analyzed neuronal area. Neuronal area was defined as NF200-positive signal within each regions of interest (ROI). For quantification purposes, ROIs were defined to achieve maximal spatial overlap with NF200-positive neuronal structures, ensuring that analyzed signals predominantly reflected neuronal compartments. All images were processed using identical thresholding parameters across all samples to ensure comparability. Representative full-field images for each experimental group are provided in Supplementary Material 1.

For GLUT4/MAP2 double immunostaining, the analysis was restricted to MAP2-positive neuronal ROIs selected from maximum intensity Z-projected images. GLUT4 immunoreactivity was assessed by measuring the mean fluorescence intensity within MAP2-positive ROIs. In addition, punctate GLUT4-positive signal was quantified using threshold-based particle analysis in ImageJ (Moments threshold), applied uniformly across all images and normalized to the analyzed MAP2-positive area.

### 2.10 Statistical analysis

The number of experimental groups and replicates is indicated in the figure legends. Data were tested for normality (Shapiro-Wilk test) and homogeneity of variances (Levene’s test). Sample sizes were selected based on previous studies (Popek et al., 2022) using the same experimental models demonstrating consistent effects. For comparisons involving three groups (two experimental groups versus one control), one-way ANOVA followed by Dunnett’s *post-hoc* test was performed. Data are presented as mean ± standard error of the mean (SEM) in all figures, with statistical significance set at *p* < 0.05. Statistical analyses were conducted using GraphPad Prism 7 (GraphPad Software Inc., San Diego, CA, United States).

## 3 Results

### 3.1 Systemic, histological, and behavioral effects of TGF-β neutralization and acute HE

**FIGURE 2:**
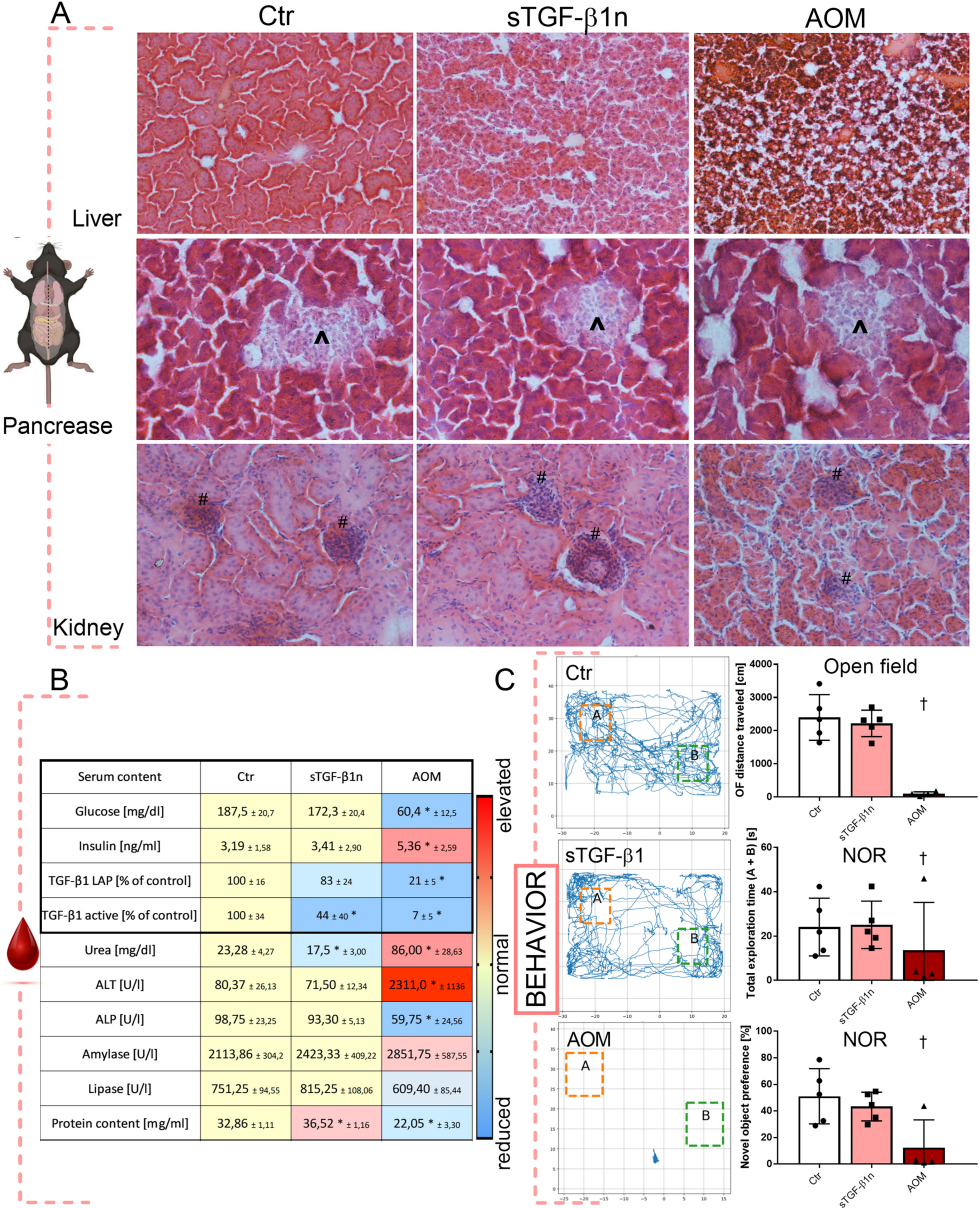
Systemic and behavioral alterations observed in control (Ctr), mice after systemic TGF-β1 neutralization (sTGF-β1n) and mice in acute hepatic encephalopathy model (AOM). **(A)** Histological staining of organs involved in glucose homeostasis—liver, pancreas, and kidney—using hematoxylin and eosin (H&E). ^∧^ indicate selected Langerhans islets in the pancreas; # mark renal glomeruli. Magnification: × 400. **(B)** Plasma biomarkers reflecting hepatic and renal function, as well as selected metabolites relevant to glucose homeostasis, were measured. ALT, alanine aminotransferase; ALP, alkaline phosphatase. *p < 0.05, One-Way ANOVA, sample sizes: insulin (*n* = 6 per group); glucose, urea, ALT, ALP, amylase, lipase, total protein (Ctr *n* = 8; sTGF-β1n and AOM *n* = 5); TGF-β1 (Ctr *n* = 5; sTGF-β1n and AOM *n* = 4). **(C)** Behawioral assessment: representative exploration tracks from the NOR test phase (A × B) are shown for each group. Open Field locomotor activity is presented as total distance traveled, while NOR performance is shown as total object exploration time (A + B) and novel object preference (%). Data are presented as mean ± SEM; *n* = 5 for Control and sTGF-β groups, and *n* = 4 for the AOM group. † excluded from inferential analysis due to profound toxin-induced hypoactivity and insufficient exploratory behavior.

Histopathological assessment of relevant tissues was carried out using standard H&E staining. In the liver of an AOM-treated animal, we observed hepatocellular necrosis and collapse of the reticulin scaffold, consistent with acute hepatic injury (Popek et al., 2025). Interestingly, neutralization of TGF-β1 in otherwise healthy controls induced subtle nuclear changes in hepatocytes, but without overt pathology. TGF-β1 has been documented in other studies as a factor that mitigates liver injury progression in hepatic diseases (Ling et al., 2013; Fabregat et al., 2016; Braczkowski et al., 2024; Wang et al., 2025). In the pancreas, no morphological abnormalities were detected following systemic TGF-β1 neutralization. In the AOM model, minor structural changes were observed in small ducts, whereas the islets of Langerhans, critical for insulin secretion, remained intact. This suggests that the peripheral insulin increase observed in AOM mice is more likely due to impaired hepatic clearance rather than intrinsic pancreatic dysfunction. In the kidney, no glomerular abnormalities were observed under either condition (Figure 2A).

However, AOM animals displayed parenchymal alterations outside the glomeruli, reminiscent of acute tubular injury reported in cirrhotic patients. These renal changes should be interpreted as secondary manifestations of systemic acute liver failure rather than direct contributors to neuronal glucose dysregulation. Together, these findings confirm that the AOM model induces multi-organ injury, while TGF-β1 neutralization primarily reproduces the reduction in circulating TGF-β1 without major extrahepatic pathology. Importantly, neither pancreatic nor renal alterations appear to directly account for the observed impairments in neuronal GLUT4 dynamics, underscoring the central role of liver-brain crosstalk in this context.

Histological findings were complemented by the analysis of systemic markers and key metabolites associated with the progression of HE in the animals’ blood serum. In the sTGF-β1n mice, no significant changes in ALT, ALP, amylase, or lipase levels were observed, which supports the absence of overt hepatic pathology, despite the somewhat ambiguous histological appearance observed in H&E staining (Figure 2B). Interestingly, serum urea levels in this group tended to reduce, aligning with longstanding scientific discourse on the multifaceted role of TGF-β1 in renal function and its potential implications in renal pathophysiology (Russ et al., 2015; Gu et al., 2020; Zhang et al., 2020; Hong et al., 2025), although no morphological changes in the kidney were observed in our model.

We confirmed a significant decrease in TGF-β1 levels, validating both the efficacy of the neutralization model and its relevance in mimicking the specific pathological feature observed in the AOM model [see (Popek et al., 2022)]. No changes in serum glucose or insulin levels were detected (Figure 2B), indicating that single-factor TGF-β1 inhibition does not alter hepato-pancreatic function.

In contrast, the mouse model of acute HE (AOM) showed marked biochemical abnormalities consistent with multi-organ dysfunction. Elevated levels of ALT, urea, and amylase, along with ALP, lipase, and total plasma proteins, decrease, correspond well with the histological liver alterations and other peripheral organs, highlighting the significant systemic nature of the AOM-induced acute liver failure (ALF) model. Additionally, both the active and latent forms of TGF-β1 were significantly reduced in AOM animals (Figure 2B). This observation, in line with previous findings (Popek et al., 2022), is likely to reflect thrombocytopenia in the AOM model, since platelets are a major source of circulating TGF-β1, even when intrahepatic production remains elevated due to liver injury.

Importantly, a significant reduction in serum glucose levels in AOM mice reflects clinical observations of hypoglycemia as a common complication associated with poor prognosis (Yang et al., 2023). Counterintuitively, in our study, hypoglycemia was accompanied by elevated peripheral insulin levels. Given the mild morphological changes observed in the pancreas, this observation is likely explained by impaired hepatic insulin clearance due to liver dysfunction, as previously reported (Elkrief et al., 2016; Najjar and Perdomo, 2019).

Taken together, the results showed liver failure in AOM-treated mice, accompanied by systemic reductions in glucose and TGF-β1 levels, along with elevated peripheral insulin concentration. In contrast, sTGF-β1n in control mice did not affect markers of liver or kidney damage, nor alter glucose and insulin levels. However, it effectively reproduced the serum TGF-β1 reduction observed in AOM animals, supporting its utility as a targeted model for studying the isolated contribution of this single factor within the otherwise complex and multifactorial pathology of acute hepatic encephalopathy.

Behavioral testing revealed profound impairment in the AOM model of acute HE, characterized by a rapid decline in locomotor activity shortly after AOM administration and progressive deterioration consistent with the symptomatic stage of disease. In accordance with this phenotype, animals exhibited minimal exploratory activity in the Open Field test and did not reach the minimal exploration threshold required for reliable interpretation of the Novel Object Recognition task, precluding quantitative assessment of memory performance (Figure 2C and Supplementary Material 1). In contrast, mice subjected to peripheral TGF-β1 neutralization showed no changes in locomotor activity or center-zone exploration in the Open Field test compared with controls. In the Novel Object Recognition task, sTGF-β1 mice showed a non-significant trend toward reduced novel object exploration, without changes in general exploratory behavior (Figure 2C and Supplementary Material 1).

### 3.2 Brain glucose metabolism and insulin-dependent glucose transport

**FIGURE 3:**
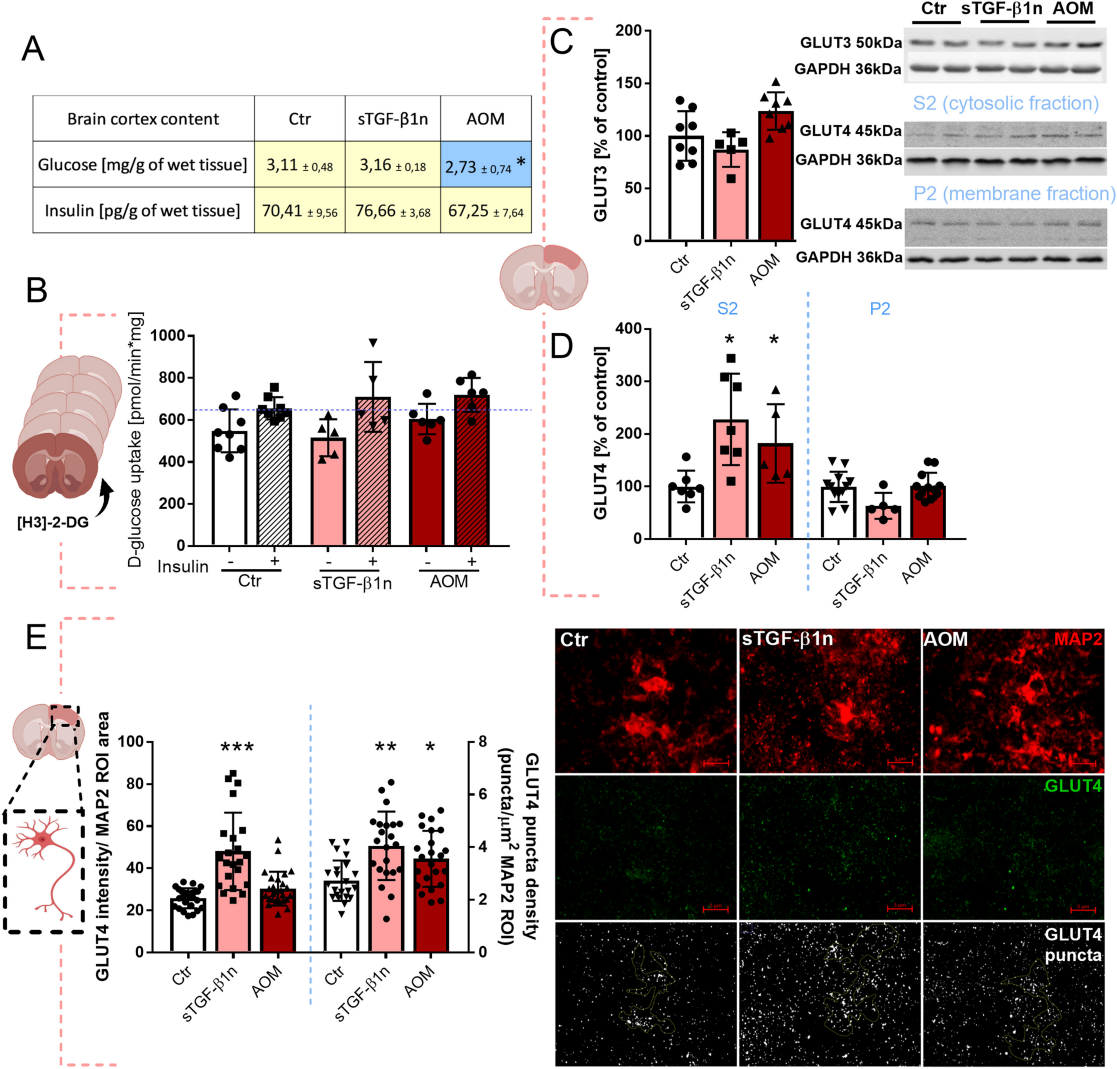
Brain metabolic profile in relation to systemic glucose metabolism and insulin-sensitive glucose transport. **(A)** Brain cortex homogenates content of glucose and insulin. *P < 0.05, One-Way ANOVA, sample sizes: insulin (*n* = 5–6); glucose (*n* = 4). **(B)** Quantification of radiolabeled D-glucose uptake in mouse brain slices with or without insulin stimulation. The blue dashed line indicates the mean level of D-glucose uptake in insulin-stimulated slices from control brains. Sample sizes: Ctr *n* = 8; sTGF-β1n *n* = 5 and AOM *n* = 6 animals per group, with five slices analyzed per animal. **(C,D)** Representative Western blots and quantification of GLUT3 protein level in the total homogenate and GLUT4 protein levels in the cytosolic (S2) and membrane (P2) fractions. *P < 0.05, One-Way ANOVA, sample sizes: GLUT3 (Ctr and AOM *n* = 8; sTGF-β1n *n* = 5) GLUT4 S2 (Ctr and sTGF-β1n *n* = 7; AOM *n* = 5) GLUT4 P2 (Ctr and AOM *n* = 12; sTGF-β1n *n* = 5). **(E)** GLUT4/MAP2 immunofluorescence showing GLUT4 signal intensity within MAP2-positive neuronal ROIs and GLUT4 puncta density normalized to MAP2 area. Representative images display MAP2 (red), GLUT4 (green), and GLUT4 puncta segmentation (ImageJ, Moments threshold). Data are shown per ROI (*n* = 3 per group; 24–26 ROIs for intensity, 22 ROIs for puncta). *p < 0.05, **p < 0.01, ***p < 0.001.

We analyzed brain sections obtained from both experimental models to investigate the status of glucose metabolism in the mouse brain, influenced by both systemic alterations and intrinsic changes within the brain tissue itself. The systemic reduction in glucose levels was reflected in the brain and represented a component of the pathophysiological status of AOM-treated mice. Specifically, glucose concentration in the frontal cortex was decreased by approximately 13%. No significant changes in cerebral insulin levels were observed across the experimental groups (Figure 3A).

In a subsequent experiment, we measured the uptake of radiolabeled D-glucose in brain slices obtained from mice in both experimental models. The results revealed a trend toward increased glucose uptake in slices from AOM-treated animals, while no changes were observed in brain slices derived from the sTGF-β1n model. In the presence of insulin, brain slices from both the sTGF-β1n and AOM models exhibited a trend toward increased D-glucose uptake, consistent with the activation of insulin-dependent pathways (Figure 3B). This effect was observed relative to insulin-treated control slices, suggesting a tendency toward enhanced efficiency of insulin-sensitive glucose transporters.

We also documented a pronounced trend toward an increase in the neuronal glucose transporter GLUT3, which stabilizes neuronal glucose uptake and utilization, in cerebral cortex homogenates from AOM mice (Figure 3C). Regarding the insulin-sensitive glucose transporter GLUT4, which facilitates additional glucose supply to neurons under conditions of elevated energy demand, we observed its accumulation in the cytosolic fraction in both the sTGF-β1n and AOM models. This cytosolic accumulation was not accompanied by a corresponding protein increase in the membrane fraction; instead, a decreasing trend in membrane-associated GLUT4 content was noted in the sTGF-β1n model (Figure 3D).

In line with the Western blot findings, immunohistochemical analysis of GLUT4/MAP2 double labeling revealed increased GLUT4 immunoreactivity within MAP2-positive neuronal ROIs in the sTGF-β1n group, accompanied by a significant increase in the number of GLUT4-positive puncta normalized to MAP2 area. In the AOM group, a non-significant trend toward increased GLUT4 fluorescence intensity within MAP2-positive ROIs was observed, whereas the number of GLUT4-positive puncta was significantly elevated (Figure 3E). These observations were consistent with the altered GLUT4 distribution detected by biochemical fractionation analysis.

### 3.3 Assessment of GLUT4-docking and insulin/TGF-β1 signaling surface proteins in the frontal cortex

**FIGURE 4:**
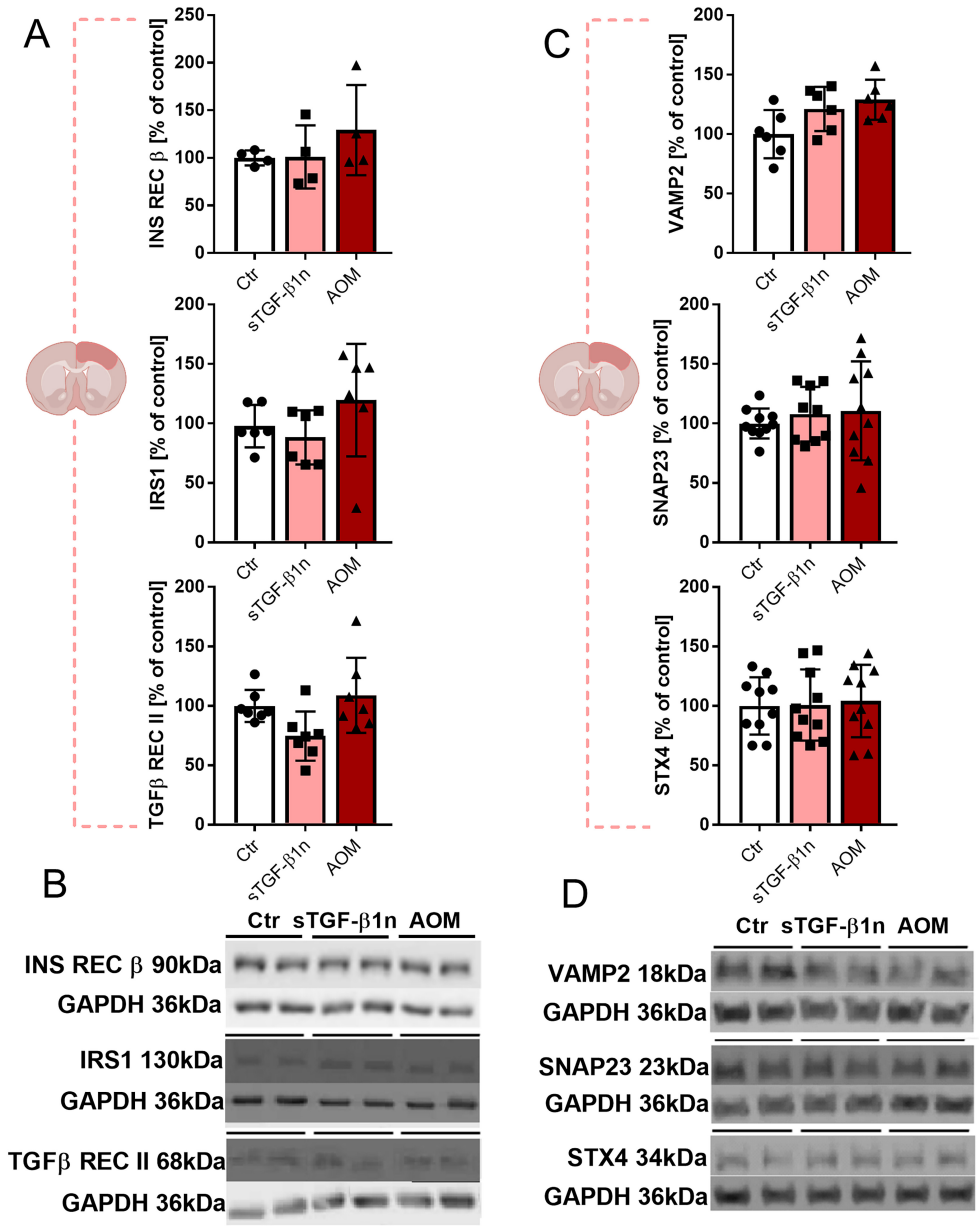
Assessment of GLUT4-docking and insulin/TGF-β1 signaling surface proteins in the frontal cortex. **(A,C)** Quantification of Insulin Receptor β (INS REC β), Insulin Receptor Substrate 1 (IRS1), TGF-β Receptor II (TGFβ REC II), and SNARE complex proteins (VAMP2, SNAP23, STX4) protein levels in the cortex homogenate. Sample sizes: INS REC β (*n* = 4); IRS1, VAMP2 (*n* = 6); TGFβ REC II (*n* = 7); SNAP23, STX4 (*n* = 10). **(B,D)** Representative Western blots.

TGFβ receptor type 2 (TGFβR2) levels in the sTGF-β1n model tended to be slightly lower, whereas the abundance of membrane-associated receptor proteins implicated in insulin- and TGF-β1-dependent signaling, namely the insulin receptor β-subunit (IRβ) and insulin receptor substrate 1 (IRS1), remained unchanged (Figures 4A,B). We then analyzed the expression of key SNARE proteins directly involved in the docking of GLUT4 to the plasma membrane, and no significant changes in the levels of VAMP2, syntaxin 4, or SNAP-25 were observed in frontal cortex homogenates across any of the experimental groups (Figures 4C,D).

### 3.4 Alterations in intracellular signaling pathways regulating GLUT4 translocation in the frontal cortex

**FIGURE 5:**
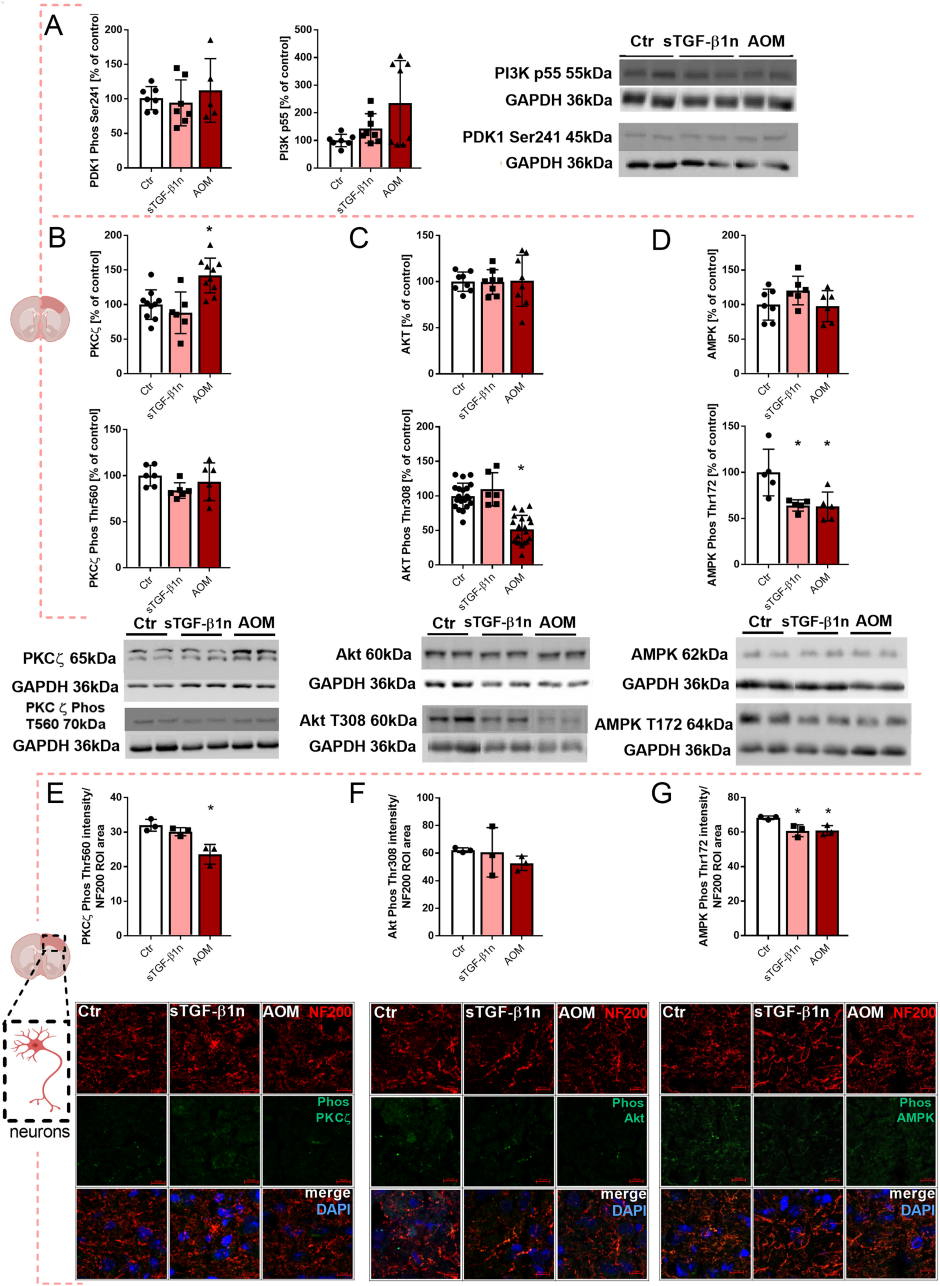
Assessment of GLUT4-docking and insulin/TGF-β1 intracellular signaling pathways in the frontal cortex. **(A–D)** Representative Western blots and quantification of PDK1 (Phos Ser241), PI3K p55, PKCζ and PKCζ (Phos Thr560), AKT and AKT (Phos Thr308), and AMPK and AMPK (Phos Thr172) protein levels in cortical homogenates. *P < 0.05, One-Way ANOVA. Sample sizes: PDK1 Phos (*n* = 5–7); PI3K p55 (*n* = 7–8); PKCζ (*n* = 6–10); PKCζ Phos (*n* = 6); AKT (*n* = 8); AKT Phos (*n* = 6–16); AMPK (*n* = 6–7); AMPK Phos (*n* = 5). **(E–G)** Microscopic analysis of PKCζ Phos Thr560, AKT Phos Thr308 and AMPK Phos Thr172 signal intensity normalized to the area of NF200-positive neurons, within regions of interest (ROIs) enriched in neuronal signal. *P < 0.05, One-Way ANOVA. Data are presented as mean values from *n* = 3 animals, with three brain sections analyzed per animal (11–19 neurons analyzed in each group, details in Supplementary Material 1).

The next analysis of receptor-activated signaling proteins involved in GLUT4 translocation revealed no statistically significant changes in the levels of phosphorylated PDK1 or PI3K in frontal cortex homogenates from either experimental model (Figure 5A). However, an increase in total PKCζ protein levels in the brains of AOM mice was accompanied by a trend toward a decrease in phosphorylation status of this protein in the sTGF-β1n group (Figure 5B). Phospho-PKCζ immunoreactivity was decreased in the frontal cortex of AOM mice, as assessed within neuronal-enriched regions identified by NF200 co-labeling (Figure 5E).

Furthermore, we detected a reduction in the phosphorylated form of Akt at Thr308 in AOM mice, with no changes in the levels of total (non-phosphorylated) Akt (Figure 5C). A similar trend toward reduced Akt activation was observed in immunohistochemical analysis using double labeling with NF200 (Figure 5F). The sTGFβ1n group showed no detectable changes in total or phosphorylated Akt levels, or in the fluorescence intensity corresponding to Phos-Akt labeling in neuronal-enriched regions (Figures 5C,F).

In parallel, reduced phosphorylation of AMPK at Thr172 was observed in both experimental models, indicating decreased AMPK activation through a PI3K/PDK1-independent pathway, without changes in total AMPK protein levels. This reduction was evident both in frontal cortex homogenates analyzed by Western blot (Figure 5D) and in cortical regions, as shown by decreased fluorescence intensity co-localizing with NF200 (Figure 5G).

## 4 Discussion

In the studied AOM model, we observed subtle morphological alterations in peripheral organs, most notably in the pancreas and kidney, beyond the brain and the liver (Figure 2A). Pancreatic morpho-architecture remained largely preserved, with only minor ductal changes and intact islets of Langerhans, indicating that documented hyperinsulinemia is more likely due to impaired hepatic clearance than intrinsic pancreatic dysfunction. Similarly, detected renal parenchymal alterations are reminiscent of acute tubular injury and consistent with the multi-organ involvement typical of acute liver failure.

While these findings reinforce the systemic nature of HE pathology, they are unlikely to account directly for the disturbances in neuronal GLUT4 trafficking described here. Instead, our results underscore the importance of focusing on the liver-brain axis, where reduced TGF-β1 availability appears to represent a critical determinant of impaired neuronal glucose handling.

In recent years, there has been growing interest in the role of TGF-β1 in different pathological conditions, including cardiovascular diseases (Zheng et al., 2017), systemic sclerosis (Lomelí-Nieto et al., 2023), idiopathic pulmonary fibrosis (Tu et al., 2024) and demyelinating diseases of the CNS (e.g., experimental models of Multiple Sclerosis) (Xie et al., 2022), those affecting the central nervous system and peripheral organs. Numerous studies highlight the pleiotropic nature of this cytokine, whose effects are highly dependent on environmental and experimental conditions.

In the context of HE, the role of TGF-β1 is typically framed within the well-characterized mechanism of hepatocyte injury, apoptosis, and necrosis, where it acts as a key pro-fibrotic and cytotoxic mediator (Fabregat et al., 2016; Chaudhary et al., 2025). Notably, recent studies in pediatric ALF patients have also reported elevated levels of TGF-β1(El-Guindi et al., 2025). Several reports have shown that peripheral inhibition of TGF-β1 can attenuate or delay liver damage, consequently mitigating the progression of HE-related neurological symptoms in animal models (McMillin et al., 2014; Raghunandhakumar et al., 2024). This approach may be beneficial in providing timing, dosage, and a broader systemic context.

However, such strategies might prove insufficient in scenarios where liver damage has progressed beyond the point of reversibility, or where additional systemic factors come into play. For instance, our previous work demonstrated the occurrence of thrombocytopenia in the AOM model of HE (Popek et al., 2022). Given that platelets are a major reservoir of TGF-β1, reportedly containing up to 40-100 times more TGF-β1 than other tissues (Assoian et al., 1983), a state of thrombocytopenia, which has also been clinically observed in HE patients (Schiødt et al., 2003; Giannini, 2006), may result in an unintended systemic reduction of TGF-β1 levels. In this context, such a reduction may act as a pathophysiological factor in itself, albeit in a direction opposite to the commonly discussed elevation of TGF-β1 in hepatic tissue or serum. Notably, previous studies have reported reduced TGF-β1 levels in serum extracellular vesicles from patients with minimal hepatic encephalopathy (Gallego et al., 2022) and decreased TGF-β1 in the serum of rats with mild liver damage (Mincheva et al., 2024), suggesting that lowered TGF-β1 in HE may result from complex systemic alterations rather than representing a uniform upregulation associated with liver injury. In contrast to reports describing increased TGF-β1 signaling in AOM-induced HE models (McMillin et al., 2019), our findings indicate a reduction of systemic TGF-β1 levels under the conditions analyzed, suggesting a more complex and potentially stage-dependent regulation of this cytokine in acute HE. The discrepancy between studies may reflect differences in the stage of disease progression at the time of sampling, as systemic complications such as thrombocytopenia may not yet be fully developed in the earlier phases of the AOM model or may develop dynamically. Alternatively, variations in experimental timing, source of circulating TGF-β1, or compensatory systemic responses may contribute to the observed differences in serum TGF-β1 levels. The consequences of systemically diminished TGF-β1 in the complex and multifactorial HE pathology therefore, warrant further characterization.

The reduction of TGF-β1 levels observed in the AOM model appears to be a secondary phenomenon, arising in the context of extensive liver damage and possibly involving dysfunction in other organs. Accordingly, the TGF-β1 neutralization model constitutes an artificial and non-physiological construct. Nonetheless, it provides a useful tool for the study of the role of a single factor, TGF-β1, within the complex pathophysiology of HE. The rationale for characterizing the anti-TGF-β1 intervention in control animals was not to assess early disease changes *per se*, but rather to determine whether TGF-β1 neutralization might itself contribute to the observed alterations in the AOM model. Furthermore, it allowed us to evaluate whether potential downstream effects of TGF-β1 deficiency might originate from peripheral disturbances—an important consideration for any future mechanistic investigations. Behavioral tests further support this distinction, as AOM-treated animals displayed severe global impairment limiting performance in exploratory tasks, whereas TGF-β1 neutralization in healthy mice did not affect general locomotor activity and minimally influenced object exploration.

The role of TGF-β1 in glucose metabolism has been addressed in various pathological contexts, including metabolic syndrome, diabetes, and neurodegenerative conditions, across different tissues (Wu and Derynck, 2009; Yadav et al., 2011; McIlvenna et al., 2021; Liu and Chen, 2022; Xu et al., 2024). This may be particularly relevant given that one of the major consequences of liver failure is impaired brain energy metabolism, significantly contributing to the development of neurological symptoms in HE. The functional overlap between TGF-β1 signaling and pathways regulating cellular energy homeostasis may provide a mechanistic link worth further exploration.

One possible interpretation of our findings is that glucose scarcity in HE may trigger a compensatory response aimed at preserving neuronal energy supply. This is supported by the observed trend toward increased levels of GLUT3, a high-affinity neuronal glucose transporter, and GLUT4, an insulin-sensitive transporter typically recruited under conditions of elevated neuronal activity and metabolic demand (Figures 3C,D). While cerebral glucose uptake reflects the coordinated activity of multiple transport systems and cell types, including GLUT1, GLUT3, and potentially non-neuronal expression of GLUT4 reported in specific brain regions (Xu et al., 2025), the present analyses combine biochemical and neuron-enriched (MAP2-positive) approaches to provide a complementary perspective on neuronal alterations without excluding broader cellular contributions. Additional GLUT4/MAP2 immunofluorescence analysis further demonstrated increased neuronal GLUT4 immunoreactivity and increased numbers of GLUT4-positive puncta within neuronal compartments in HE animals (Figure 3E). While the increased intracellular accumulation of GLUT4 may reflect enhanced production and/or vesicular retention as part of this compensatory response, our data suggest impairment or inefficient GLUT4 mobilization toward the plasma membrane, although direct assessment of membrane-associated pools was not analyzed. Interestingly, despite the ongoing debate and lack of clarity regarding the relative contribution of peripherally derived versus locally synthesized insulin in the brain (Milstein and Ferris, 2021), no significant changes in cerebral insulin levels were observed across the experimental groups (Figure 3A).

Previously, we reported that presynaptic SNARE proteins involved in neurotransmitter vesicle docking are impaired in the AOM model (Popek et al., 2018). However, the SNARE machinery mediating GLUT4 vesicle docking represents a distinct subset of SNAREs, including VAMP2, syntaxin-4, and SNAP-25 (Bryant and Gould, 2011). In the present study, we found no significant alterations in their levels, suggesting that the defect in GLUT4 translocation may not occur at this stage of the fusion process. Similarly, membrane-associated insulin receptor components (IRβ and IRS1) showed no significant changes. Although systemic insulin levels are elevated in this model, central insulin signaling appears to remain largely preserved, at least in terms of receptor abundance (Figures 4A–D).

Interestingly, key signaling proteins that promote GLUT4 translocation, such as PKCζ and Akt (Van Dam et al., 2005; Liu et al., 2007; Luiken et al., 2009), exhibited dysregulated phosphorylation patterns in the AOM model. Notably, Akt activation appeared reduced, whereas PKCζ showed model-dependent alterations in total and phosphorylated forms (Figures 5B,C,E,F), indicating that these kinases respond differently to systemic and metabolic disturbances. Such divergence may contribute to the cytosolic retention of GLUT4 observed in AOM mice and likely reflects mechanisms beyond direct TGF-β1 signaling. Given its sensitivity to multiple upstream cues, PKCζ may function as a transient modulator rather than a stable determinant of GLUT4 translocation, consistent with its proposed role in linking insulin and AMPK pathways, as demonstrated in peripheral tissues (Liu et al., 2010; Habets et al., 2012).

A particularly noteworthy observation, however, is the decrease in AMPK phosphorylation at Thr172 in both experimental models (Figures 5D,G). Given AMPK’s well-established role as a key metabolic regulator of GLUT4 translocation and cellular energy sensing across different tissues (Witczak et al., 2008; Ashrafi et al., 2017; Niu et al., 2017), this pathway may represent a mechanistic link between TGF-β1 signaling and neuronal energy metabolism. Previous reports indicate reciprocal regulation between TGF-β1 and AMPK, where TGF-β1 can modulate AMPK activity, and AMPK in turn can influence TGF-β1 signaling (Thakur et al., 2015; Gao et al., 2018; Liu et al., 2018; Pan et al., 2018). These findings suggest that TGF-β1 deficiency may contribute to impaired GLUT4 translocation through altered AMPK activity.

Our study is not without limitations, the most notable being the non-fully mechanistic nature of the findings obtained from the two experimental models. TGF-β1 signaling may exert context-dependent effects, varying with the specific cellular and systemic environment. Moreover, the observed alterations in neuronal glucose handling are likely influenced by multiple interacting factors and cannot be attributed exclusively to reduced TGF-β1 availability. Thus, the interpretations presented here represent one possible scenario among several. Importantly, our results highlight the potential role of underappreciated systemic factors as modulators or amplifiers of HE-related deficits, emphasizing the need to broaden the scope of future investigations.

Taken together, our findings suggest that the accumulation of cytosolic GLUT4 observed in HE may represent a compensatory response to reduced glucose availability in metabolically stressed neurons. However, impaired or inefficient GLUT4 mobilization toward the plasma membrane may limit this adaptation, potentially contributing to neuronal energy crisis. In the case of systemic TGF-β1 neutralization, GLUT4 accumulation may reflect altered GLUT4 mobilization dynamics, potentially involving impaired AMPK signaling. These results suggest a possible, previously underappreciated role of TGF-β1 in the control of neuronal energy metabolism in HE.

## Data Availability

The original contributions presented in this study are included in this article/Supplementary material, further inquiries can be directed to the corresponding author.
