# Supplementary material for: Systemic TGF-β1 reduction contributes to neuronal GLUT4 trafficking impairment in acute hepatic encephalopathy
Source: Front Mol Neurosci. 2026 Jun 10;19:1849470. doi: 10.3389/fnmol.2026.1849470 (PMC13291138; doi:10.3389/fnmol.2026.1849470)
Supplement: Supplementary file 1 [file Data_Sheet_1.docx]

**Supplementary materials to**
*Systemic TGF-β1 reduction contributes to neuronal GLUT4 trafficking impairment in acute hepatic encephalopathy*
M. Popek¹*, M. Zielińska¹

**Supplementary Methods and Figures**

- **S1–S2 and Table S1**: Description and documentation of insulin quantification in mouse brain homogenates.
- **S3-S4:** Description of supplementary behavioral assessment parameters.
- **S5**: Analysis of fluorescence intensity in individual neuronal regions of interest (ROIs).
- **S6–S8**: High-resolution immunohistochemical images corresponding to the smaller images presented in Main Figure 5E–G.
- **S9**: Secondary antibody-only control stainings
- **S10–S26**: Representative raw Western blot images demonstrating protein band quality.

Note: Some bands are repeated from those shown in the main figures; here, full-length images are presented without cropping and including all samples, rather than limiting the number of samples (“n”), to ensure transparency and document the full dataset.

***Description of insulin quantification in mouse brain homogenates.***

For the measurement of insulin levels in mouse brain homogenates, an Mouse Insulin ELISA Kit (cat. no. KE10089; Proteintech Group Inc., Rosemont, IL, USA) was used. The assay was performed on: 5 mice for Ctr and sTGB-β1n; 4 mice for AOM.

| **Sample** | **Baseline insulin (pg/mL)** | **Spike added (pg/mL)** | **Expected (pg/mL)** | **Measured (pg/mL)** | **Recovery (%)** |
| --- | --- | --- | --- | --- | --- |
| Cortex + low spike | 2,28 | 12,5 | 14,78 | 14,43 | 97,63 |
| Cortex + high spike | 2,28 | 50 | 52,28 | 47,16 | 90,21 |
|  | **Measured (pg/mL)** | **dilution-corrected values** | **Relative recovery (%)** |  |  |
| Serum K5 1:50 | 45,215 | 2260,75 | 94,04 |  |  |
| Serum K5 1:100 | 24,041 | 2404,1 | 106,34 |  |  |

**Supplementary Table S1**. Spike-and-recovery and dilution linearity of insulin ELISA measurements


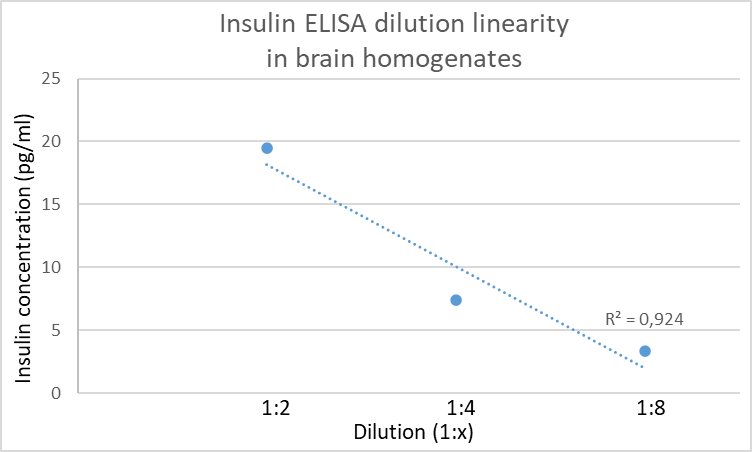


**Supplementary Figure S1**. dilution linearity of insulin ELISA measurements in brain homogenates. The dilution series showed acceptable linearity (R² = 0.924).


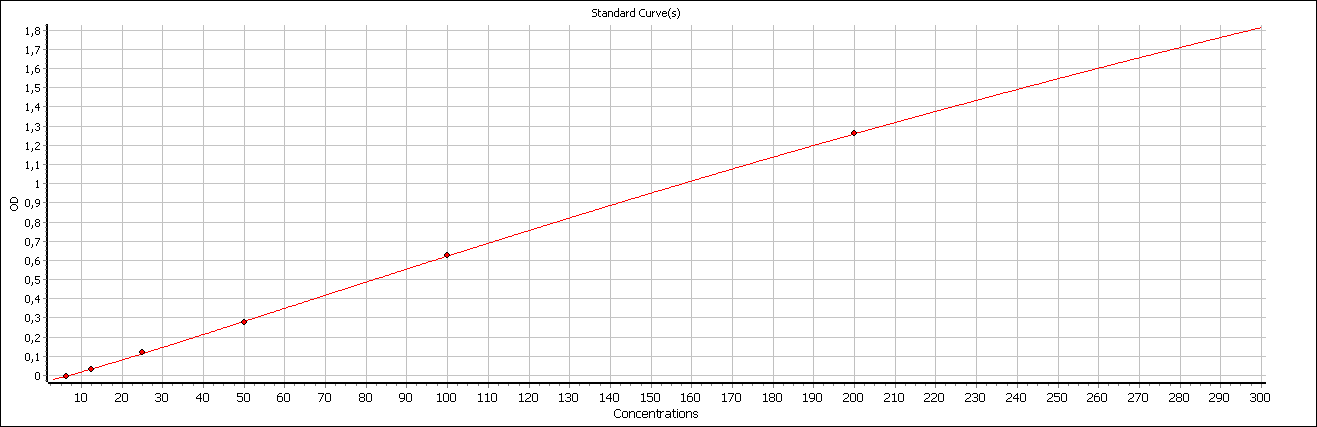


**Supplementary Figure S2**. Insulin ELISA standard curve fitted with a four-parameter logistic (4PL) model. The curve demonstrated excellent fit (R² = 0,99993).

### ***Description of supplementary behavioral assessment parameters.***

### **Open Field**

**Supplementary Figure S3.** Time spent in the center zone during the Open Field test [s]

**Novel Object Recognition - familiarization phase (AxA’)**

**Supplementary Figure S4.** Total and object-specific exploration time (A + A′; A vs A′) during the familiarization phase [s]

### ***Analysis of fluorescence intensity in individual neuronal regions of interest (ROIs).***

This figure presents raw fluorescence intensity data measured for individual neuronal ROIs, rather than the averaged values shown in the main manuscript. The plots illustrate the distribution of fluorescence signal for proteins phospho-AMPKα (Thr172), phospho-Akt (Thr308) or phospho-PKCζ (Thr560) in all analyzed regions across experimental groups. Each data point corresponds to a single measurement within a defined neuronal area. These data demonstrate the variability of signal intensity within and between samples, complementing the mean values reported in the main Figure 2.


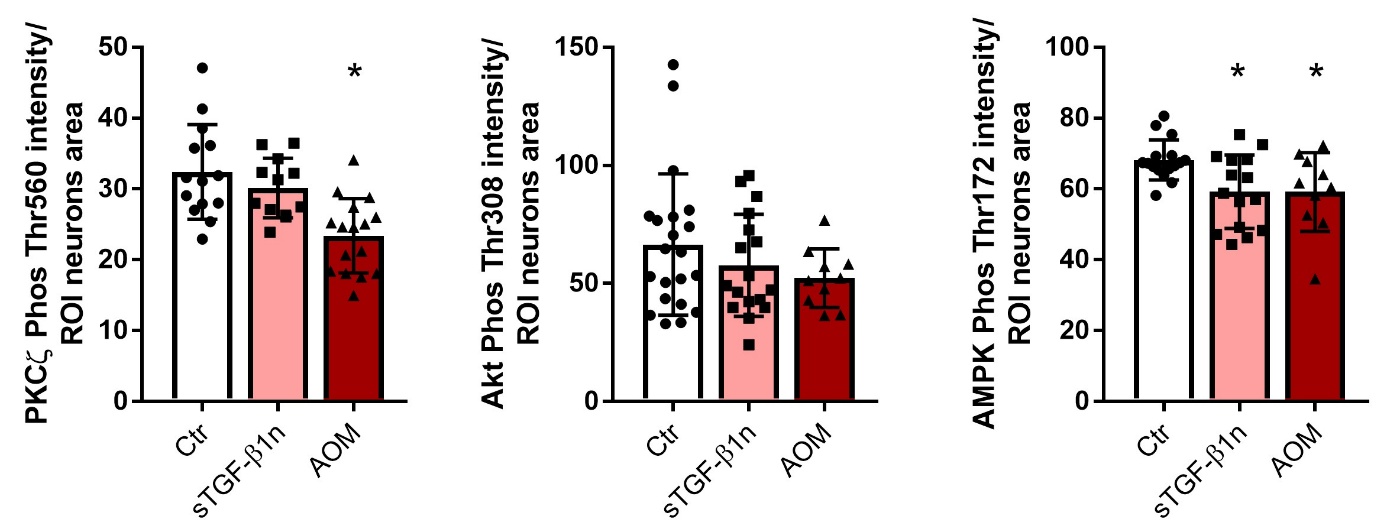


**Supplementary Figure S5.** Fluorescence intensity distribution for phospho-AMPKα (Thr172), phospho-Akt (Thr308), and phospho-PKCζ (Thr560) in individual neuronal regions of interest (ROIs) across experimental groups.


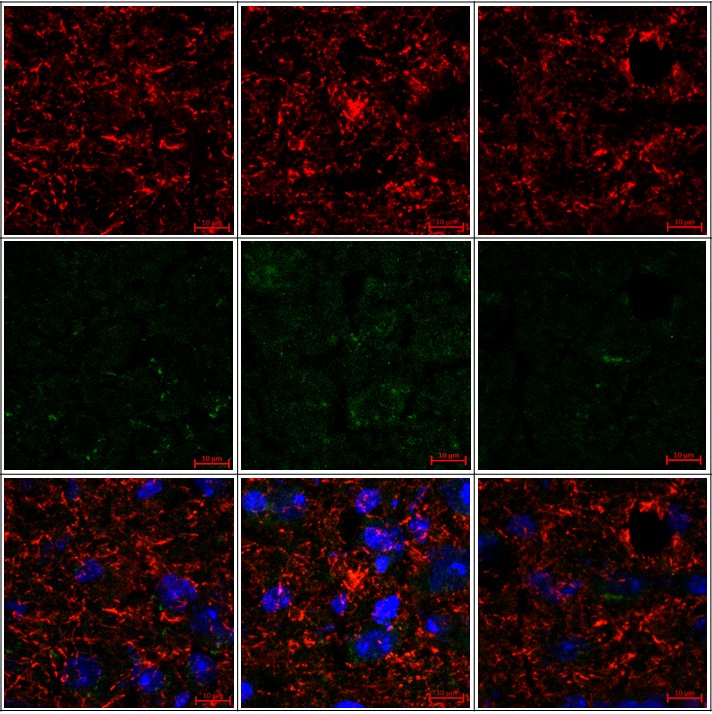


**Supplementary Figure S6.** Representative immunofluorescent staining of the frontal cortex in the studied groups (from left to right: Control, sTGFβ1n, AOM). Neurons are labeled with NF200 (red), phosphorylated PKCζ (Thr560) (green), and nuclei are counterstained with DAPI (blue). Scale bar = 10 μm.


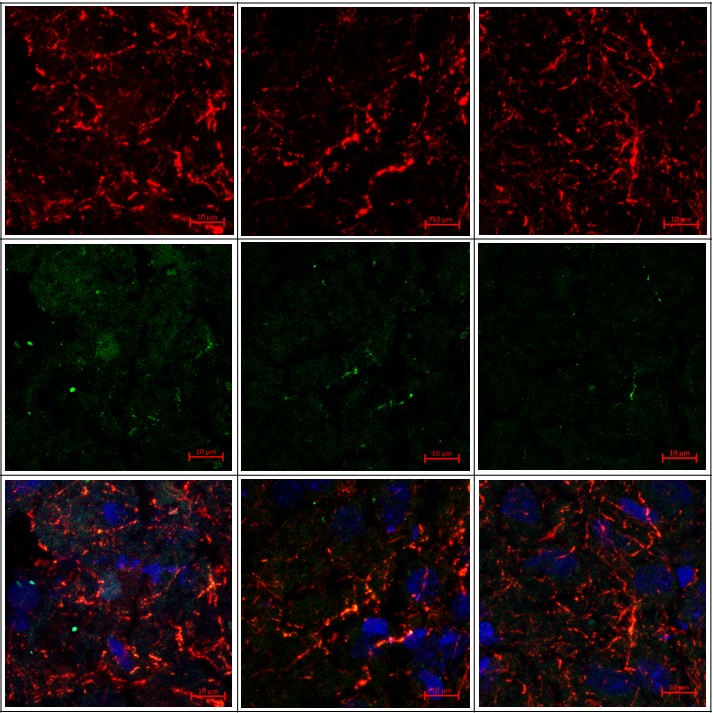


**Supplementary Figure S7.** Representative immunofluorescent staining of the frontal cortex in the studied groups (from left to right: Control, sTGFβ1n, AOM). Neurons are labeled with NF200 (red), AKT Phos Thr308 (green), and nuclei are counterstained with DAPI (blue). Scale bar = 10 μm.


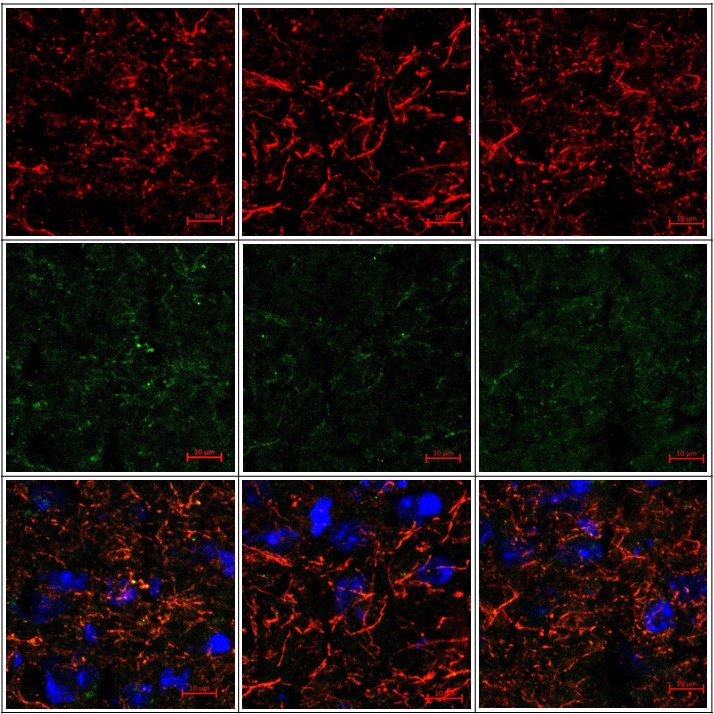


**Supplementary Figure S8.** Representative immunofluorescent staining of the frontal cortex in the studied groups (from left to right: Control, sTGFβ1n, AOM). Neurons are labeled with NF200 (red), AMPK Phos Thr172 (green), and nuclei are counterstained with DAPI (blue). Scale bar = 10 μm.


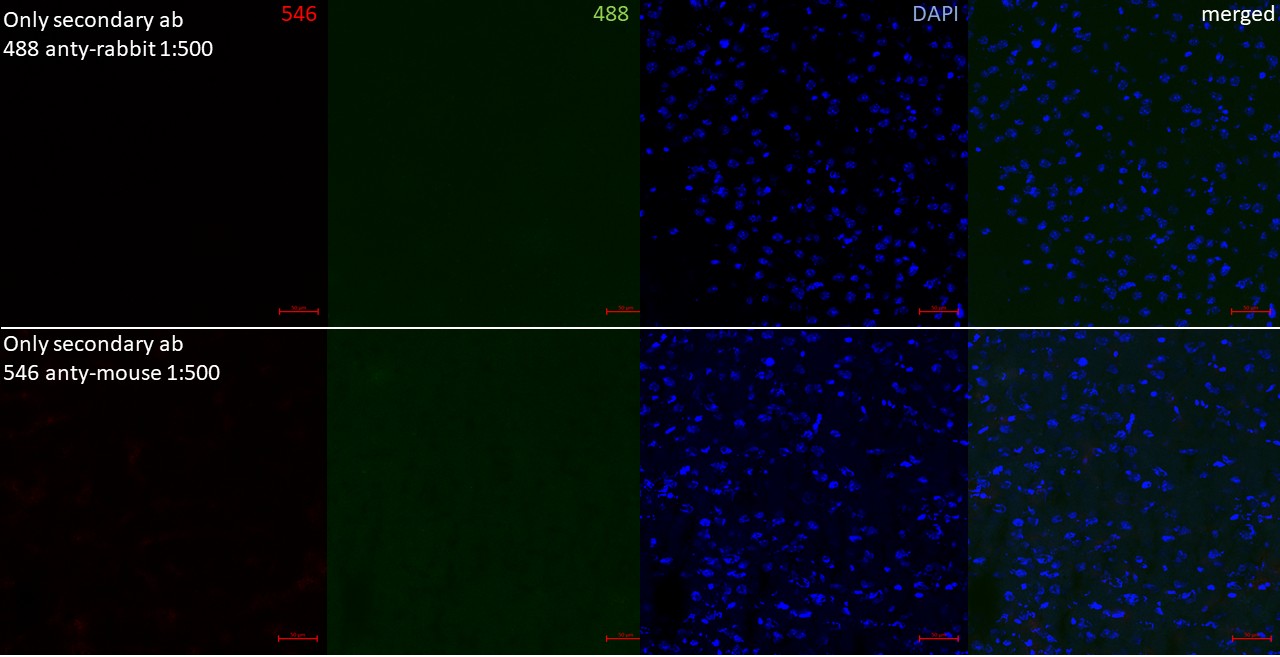


**Supplementary Figure S9.** Secondary antibody-only control stainings acquired using identical imaging settings (Alexa Fluor 488 anti-rabbit and Alexa Fluor 546 anti-mouse channels) showing minimal background fluorescence.

***Representative raw Western blot images demonstrating protein band quality.***


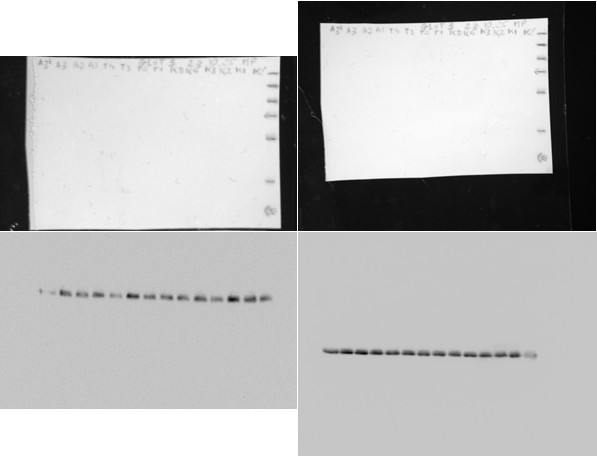


**Supplementary Figure S10.** The upper panel shows the molecular weight marker, and the lower panel presents the chemiluminescent signal obtained after incubation with: on the left - the **anti-phospho-AMPK** (Thr172, clone 40H9; 1:1000, cat. no. 2535) antibody (predicted band size: ~64 kDa); on the right - corresponding GAPDH signal detected on the same membrane after stripping, serving as a loading control. The order of cortical homogenate samples from right to left (starting from the molecular weight marker) was as follows: six control samples, four sTGF-β1n samples, and four AOM samples.


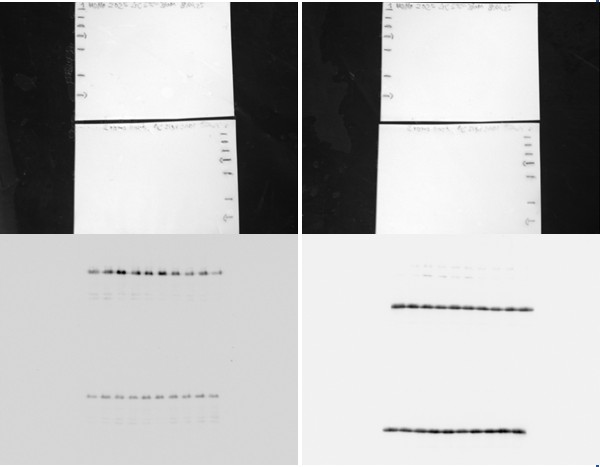


**Supplementary Figure S11.** The upper panel shows the molecular weight marker, and the lower panel presents the chemiluminescent signal obtained after incubation with: on the left - the **anti-AMPKα** (1:1000, cat. no. 5831) antibody (predicted band size: ~62kDa); on the right - corresponding GAPDH signal detected on the same membrane after stripping, serving as a loading control. The order of cortical homogenate samples (starting from the molecular weight marker) was as follows: four control samples, three sTGF-β1n samples, and three AOM samples, in repetition.


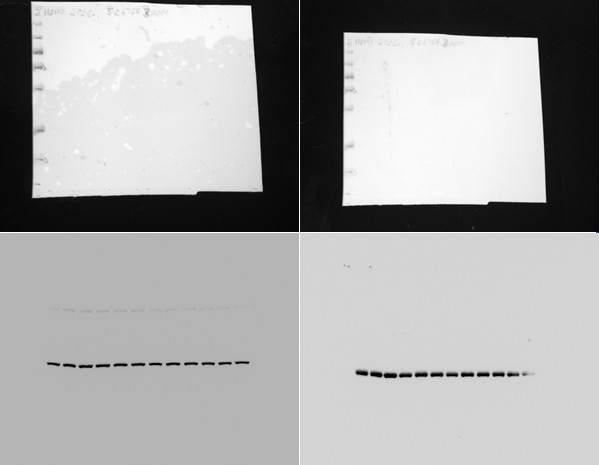


**Supplementary Figure S12.** The upper panel shows the molecular weight marker, and the lower panel presents the chemiluminescent signal obtained after incubation with: on the left - the **phospho-PDK1 (**Ser241**,** clone C49H2; 1:1000, cat. no. 3438) antibody (predicted band size: ~60 kDa, observed ~45kDa); on the right - corresponding GAPDH signal detected on the same membrane after stripping, serving as a loading control. The order of cortical homogenate samples (starting from the left - from molecular weight marker) was as follows: five control samples, four sTGF-β1n samples, and three AOM samples.


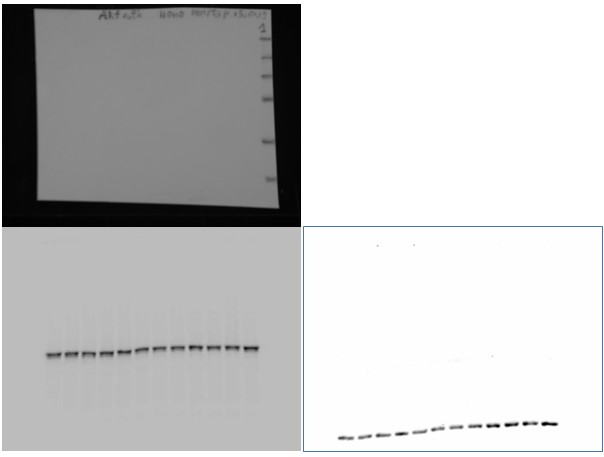


**Supplementary Figure S13.** The upper panel shows the molecular weight marker, and the lower panel presents the chemiluminescent signal obtained after incubation with: on the left - the **Akt** (1:1000, cat. no. 4691) antibody (observed band size: ~60 kDa); on the right - corresponding GAPDH signal detected on the same membrane after stripping, serving as a loading control. The order of cortical homogenate samples (starting from the right - from molecular weight marker) was as follows: four control samples, four sTGF-β1n samples, and four AOM samples.


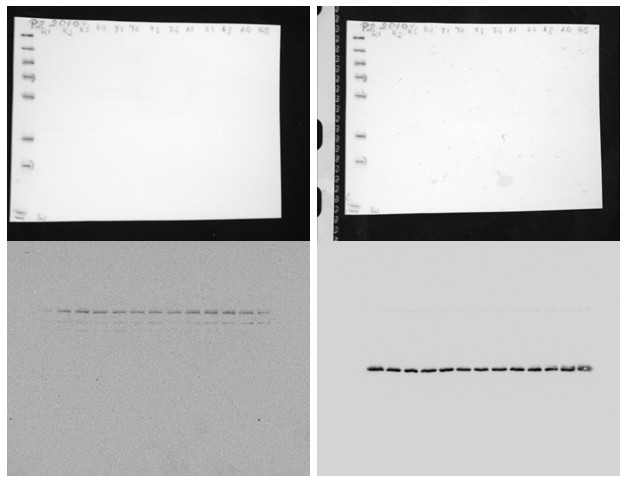


**Supplementary Figure S14.** The upper panel shows the molecular weight marker, and the lower panel presents the chemiluminescent signal obtained after incubation with: on the left - the **GLUT4** (1:750, cat. no. SAB4300667) antibody (observed band size: ~45 kDa); on the right - corresponding GAPDH signal detected on the same membrane after stripping, serving as a loading control. The order of cortical membrane (P2) fraction samples (starting from the left - from molecular weight marker) was as follows: four control samples, four sTGF-β1n samples, and five AOM samples.


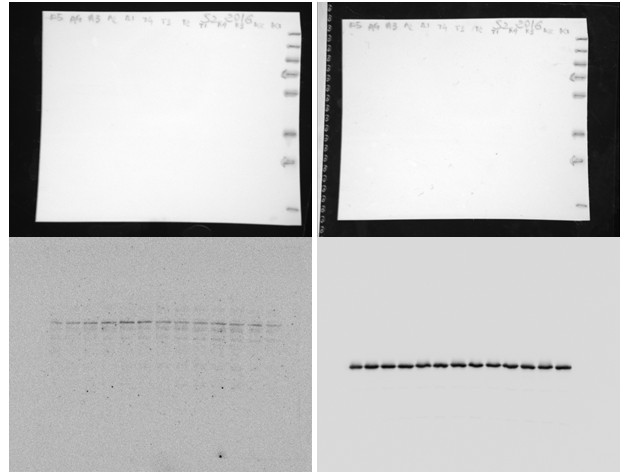


**Supplementary Figure S15.** The upper panel shows the molecular weight marker, and the lower panel presents the chemiluminescent signal obtained after incubation with: on the left - the **GLUT4** (1:750, cat. no. SAB4300667) antibody (observed band size: ~45 kDa); on the right - corresponding GAPDH signal detected on the same membrane after stripping, serving as a loading control. The order of cortical cytosol (S2) fraction samples (starting from the right - from molecular weight marker) was as follows: four control samples, four sTGF-β1n samples, and five AOM samples.


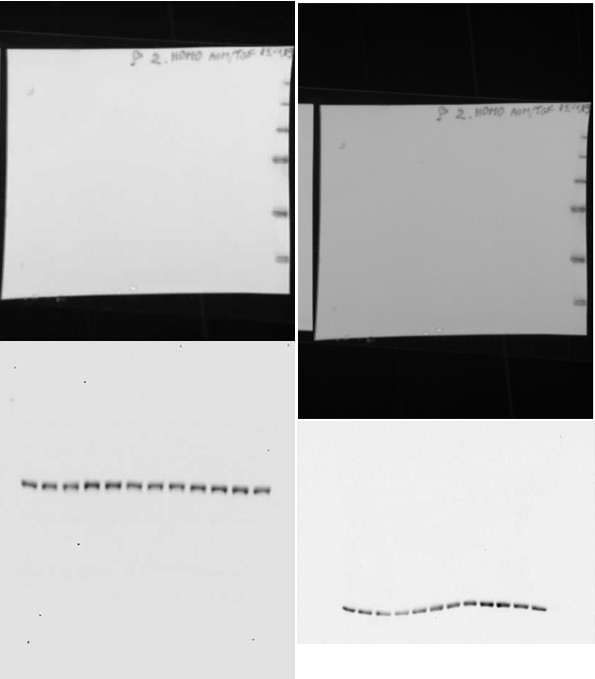


**Supplementary Figure S16.** The upper panel shows the molecular weight marker, and the lower panel presents the chemiluminescent signal obtained after incubation with: on the left - the **InsRβ** (1:300, cat. no. sc-57342) antibody (observed band size: ~90 kDa); on the right - corresponding GAPDH signal detected on the same membrane after stripping, serving as a loading control. The order of cortical homogenate samples (starting from the right - from molecular weight marker) was as follows: four control samples, four sTGF-β1n samples, and four AOM samples.


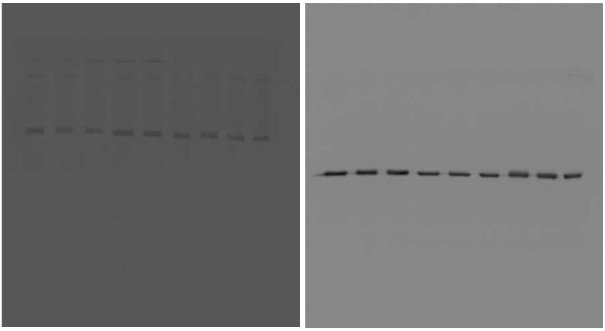


**Supplementary Figure S17.** The panel presents the chemiluminescent signal obtained after incubation with: on the left - **Anti-IRS1** (1:2000, cat. no. 06-248) antibody (predicted band size: 160kDa, observed band size: ~130 kDa); on the right - corresponding GAPDH signal detected on the same membrane after stripping, serving as a loading control. The order of cortical homogenate samples (starting from the left - from molecular weight marker) was as follows: four control samples, three sTGF-β1n samples, and three AOM samples.


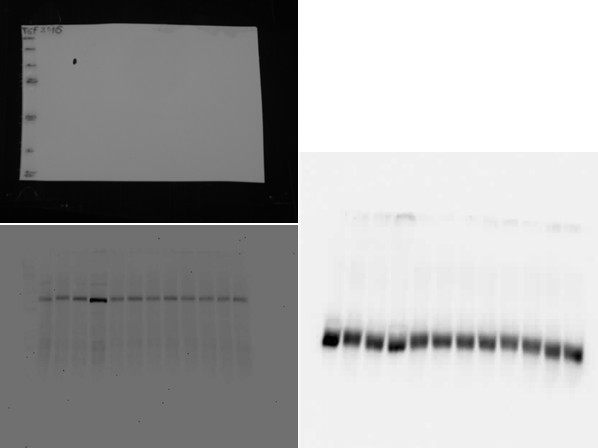


**Supplementary Figure S18.** The upper panel shows the molecular weight marker, and the lower panel presents the chemiluminescent signal obtained after incubation with: on the left - the **PI3 kinase p55** (clone D2B3; 1:1000, cat. no. 11889S) antibody (observed band size: ~55 kDa); on the right - corresponding GAPDH signal detected on the same membrane after stripping, serving as a loading control. The order of cortical homogenate samples (starting from the left - from molecular weight marker) was as follows: four control samples, four sTGF-β1n samples, and four AOM samples.


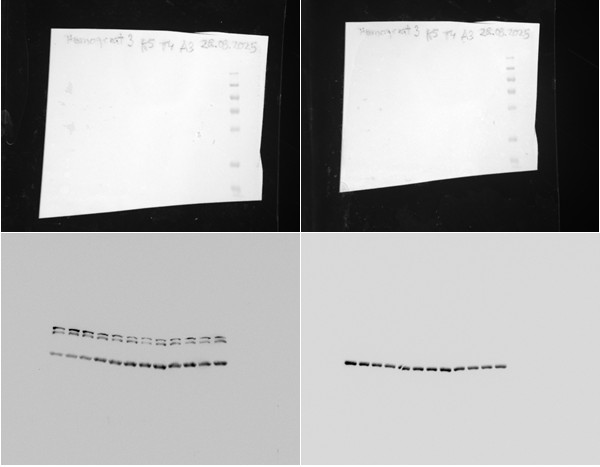


**Supplementary Figure S19.** The upper panel shows the molecular weight marker, and the lower panel presents the chemiluminescent signal obtained after incubation with: on the left - the **PKCζ** (1:400, cat. no. sc-216) antibody (predicted band size: 80kDa, observed band size: ~65 kDa); on the right - corresponding GAPDH signal detected on the same membrane after stripping, serving as a loading control. The order of cortical homogenate samples (starting from the right - from molecular weight marker) was as follows: four control samples, four sTGF-β1n samples, and four AOM samples.


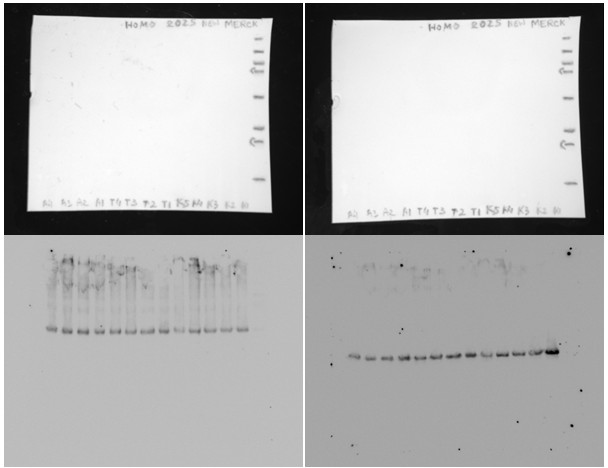


**Supplementary Figure S20.** The upper panel shows the molecular weight marker, and the lower panel presents the chemiluminescent signal obtained after incubation with: on the left - the **PKCζ PhosT560** (1:1000, cat. no. ab62372) antibody (predicted band size: 68kDa, observed band size: ~60 kDa); on the right - corresponding GAPDH signal detected on the same membrane after stripping, serving as a loading control. The order of cortical homogenate samples (starting from the right - from molecular weight marker) was as follows: five control samples, four sTGF-β1n samples, and four AOM samples.


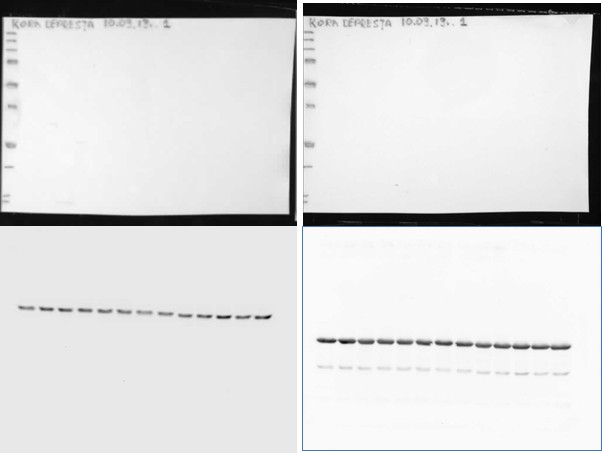


**Supplementary Figure S21.** The upper panel shows the molecular weight marker, and the lower panel presents the chemiluminescent signal obtained after incubation with: on the left - the **GLUT3** (1:2000, cat. no. ab41525) antibody (observed band size: ~50 kDa); on the right - corresponding GAPDH signal detected on the same membrane after stripping, serving as a loading control. The order of cortical homogenate samples (starting from the left - from molecular weight marker) was as follows: five control samples, four sTGF-β1n samples, and four AOM samples.


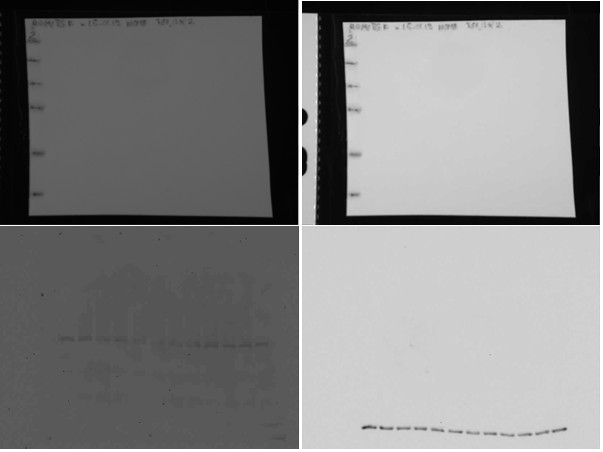


**Supplementary Figure S22.** The upper panel shows the molecular weight marker, and the lower panel presents the chemiluminescent signal obtained after incubation with: on the left - the **TGFBR2** (1:150, clone E-6; cat. no. sc-17792) antibody (observed band size: ~68 kDa); on the right - corresponding GAPDH signal detected on the same membrane after stripping, serving as a loading control. The order of cortical homogenate samples (starting from the left - from molecular weight marker) was as follows: four control samples, four sTGF-β1n samples, and four AOM samples.


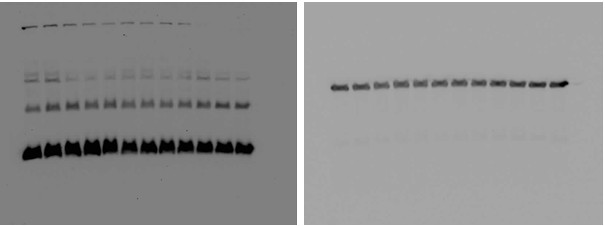


**Supplementary Figure S23.** The panel presents the chemiluminescent signal obtained after incubation with: on the left - **SNAP23** (1:500, cat. no. AB3340) antibody (observed band size: ~23 kDa); on the right - corresponding GAPDH signal detected on the same membrane after stripping, serving as a loading control. The order of cortical homogenate samples (starting from the right - from molecular weight marker) was as follows: four control samples, four sTGF-β1n samples, and four AOM samples.


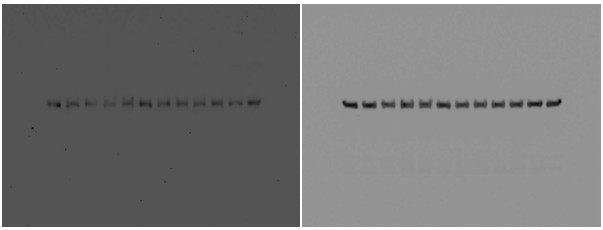


**Supplementary Figure S24.** The panel presents the chemiluminescent signal obtained after incubation with: on the left - **Syntaxin 4** (1:2000, STX4 (cat. no. AB184545) antibody (observed band size: ~34 kDa); on the right - corresponding GAPDH signal detected on the same membrane after stripping, serving as a loading control. The order of cortical homogenate samples (starting from the left - from molecular weight marker) was as follows: four control samples, four sTGF-β1n samples, and four AOM samples.


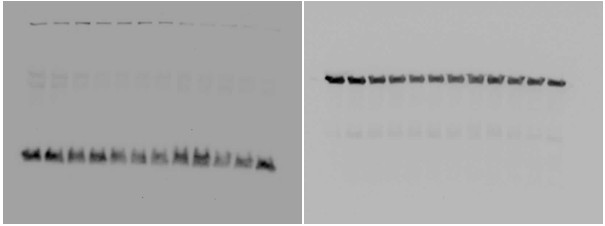


**Supplementary Figure S25.** The panel presents the chemiluminescent signal obtained after incubation with: on the left – **VAMP2 (**1:1000, cat. no. AB3347) antibody (observed band size: ~15-20 kDa); on the right - corresponding GAPDH signal detected on the same membrane after stripping, serving as a loading control. The order of cortical homogenate samples (starting from the left - from molecular weight marker) was as follows: four control samples, four sTGF-β1n samples, and four AOM samples.


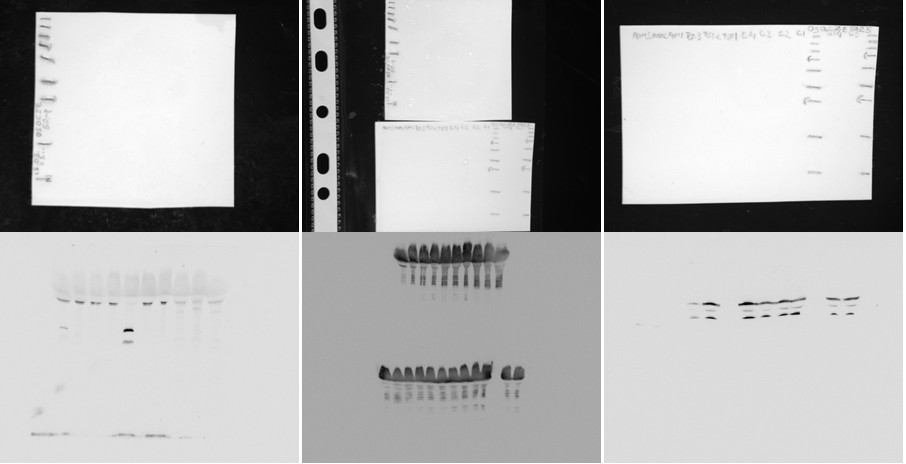


**Supplementary Figure S26.** The panel presents the chemiluminescent signal obtained **in serum** after incubation with: on the left – **TGF-β1 (**1:750, cat. no. 26155-1-AP) antibody (observed band size: ~45 kDa); in the middle - corresponding Albumine (1:5000, cat. no. ab207327) signal detected on the same membranes after stripping, serving as a loading control; on the right – **TGF-β1** (1:2000; cat. no. AF-101-NA), (observed band size: ~40 kDa and ~25kDa). The order of serum samples (starting from molecular weight marker) was as follows: for the left panel: four control samples, three sTGF-β1n samples, and three AOM samples; for right panel: one control and one sTGF-β1n followed by four control, three sTGF-β1n samples, and three AOM samples.
